# A comprehensive tool for creating and evaluating privacy-preserving biomedical prediction models

**DOI:** 10.1186/s12911-020-1041-3

**Published:** 2020-02-11

**Authors:** Johanna Eicher, Raffael Bild, Helmut Spengler, Klaus A. Kuhn, Fabian Prasser

**Affiliations:** 1 School of Medicine, Technical University of Munich, Ismaninger Str. 22, Munich, 81675 Germany; 2 Berlin Institute of Health (BIH), Anna-Louisa-Karsch-Straße 2, Berlin, 10178 Germany; 3 Charité – Universitätsmedizin Berlin, Charitéplatz 1, Berlin, 10117 Germany

**Keywords:** Biomedical data, Prediction models, Machine learning, Classification, Privacy protection, Data anonymization

## Abstract

**Background:**

Modern data driven medical research promises to provide new insights into the development and course of disease and to enable novel methods of clinical decision support. To realize this, machine learning models can be trained to make predictions from clinical, paraclinical and biomolecular data. In this process, privacy protection and regulatory requirements need careful consideration, as the resulting models may leak sensitive personal information. To counter this threat, a wide range of methods for integrating machine learning with formal methods of privacy protection have been proposed. However, there is a significant lack of practical tools to create and evaluate such privacy-preserving models. In this software article, we report on our ongoing efforts to bridge this gap.

**Results:**

We have extended the well-known ARX anonymization tool for biomedical data with machine learning techniques to support the creation of privacy-preserving prediction models. Our methods are particularly well suited for applications in biomedicine, as they preserve the truthfulness of data (e.g. no noise is added) and they are intuitive and relatively easy to explain to non-experts. Moreover, our implementation is highly versatile, as it supports binomial and multinomial target variables, different types of prediction models and a wide range of privacy protection techniques. All methods have been integrated into a sound framework that supports the creation, evaluation and refinement of models through intuitive graphical user interfaces. To demonstrate the broad applicability of our solution, we present three case studies in which we created and evaluated different types of privacy-preserving prediction models for breast cancer diagnosis, diagnosis of acute inflammation of the urinary system and prediction of the contraceptive method used by women. In this process, we also used a wide range of different privacy models (k-anonymity, differential privacy and a game-theoretic approach) as well as different data transformation techniques.

**Conclusions:**

With the tool presented in this article, accurate prediction models can be created that preserve the privacy of individuals represented in the training set in a variety of threat scenarios. Our implementation is available as open source software.

## Background

The digitalization of healthcare promises to enable personalized and predictive medicine [1]. Based on digital data that characterize patients and probands at comprehensive depth and breadth [2], machine learning models can be created that are able to detect unknown relationships between biomedical parameters and enable decision support systems by using the knowledge about such relationships to infer or predict parameters (henceforth called *target variables*), e.g. diagnoses or outcomes [3]. However, in such data-driven environments, it is becoming increasingly challenging to protect the data used for creating such models from privacy breaches [4]. Data privacy involves ethical, legal and societal aspects [5] and different layers of protection mechanisms must therefore be implemented [6, 7].

On the technical level, current efforts in the area of machine learning for health data put a significant focus on distributed learning which overcomes the need to share data across institutional boundaries to create the large datasets needed for training purposes [8, 9]. Cryptographic secure multiparty computation approaches are an important technique in this context [10]. Although this solves some of the privacy issues, it is important to realize that privacy protection must be addressed on multiple levels, including the output data level where it must be ensured that the resulting prediction models cannot be used to extract personal information [11]. Prediction models, which learn from anonymized data are a common solution to this problem. The core concept behind data anonymization is to transform data in such a manner that privacy risks are reduced while the reduction of risks is balanced against a reduction of data utility [12, 13]. Several high-profile re-identification attacks have shown that simply removing all directly identifying attributes (e.g. names and addresses) is not sufficient for this purpose [14, 15]. Laws and regulations, e.g. the Privacy Rule of the U.S. Health Insurance Portability and Accountability Act (HIPAA) [16] or the European General Data Protection Regulation [17], define different approaches to address this issue.

In recent years, several easy-to-use tools have been developed that make methods of data anonymization available to a broad range of users. At the same time, various methods for addressing output data privacy in machine learning have been proposed by the research community, but robust implementations that can be applied in practice are lacking. In this article, we report on our ongoing efforts to bring both worlds together by integrating machine learning techniques into a well-known data anonymization tool. In prior work, we have laid the groundwork for the results presented in this article by (1) implementing a method into the tool that ensures that anonymized output data is suitable as training data for creating prediction models, and (2) integrating logistic regression models into the tool in such a way that they can be used to assess the performance of models created from anonymized data [18]. In this software article, we present a wide range of enhancements that significantly broaden the applicability of the approach. In detail, we

1. added a method to make anonymized output data suitable for the training of multiple models that can predict different target variables,
2. implemented additional types of prediction models to enable assessing the performance of different types of privacy-preserving machine learning techniques,
3. integrated the approach with further anonymization methods, including differential privacy, which is a state-of-the-art approach offering strong privacy protection,
4. implemented a wide range of additional metrics and visualizations for assessing the impact of privacy protection on prediction performance,
5. added support for further data transformation techniques, such as data aggregation.

The resulting tool is highly versatile, as it supports binomial and multinomial target variables, different types of prediction models and a wide range of methods of privacy protection. Moreover, all techniques have been integrated into a sound framework that supports the creation, evaluation and refinement of models through intuitive graphical user interfaces. We demonstrate the broad applicability of our approach by creating different types of privacy-preserving models for breast cancer diagnosis, diagnosis of acute inflammation of the urinary system and prediction of the contraceptive method used by women using different anonymization and prediction techniques. The results show that accurate prediction models can be created that preserve privacy in a variety of threat scenarios. Our implementation is available as open source software.

## Implementation

The software described in this article has been developed by extending ARX, an open source anonymization tool which has specifically been designed for applications to biomedical data [19]. In this section, we will focus on the two most important functionalities implemented, which are (1) methods for the automated creation of privacy-preserving prediction models and (2) methods for evaluating and fine-tuning the resulting models. In the individual sections, we will describe how we addressed particularly complex challenges.

### Methods for creating privacy-preserving prediction models

In predictive modeling, the goal is to predict the value of a predefined *target variable* from a given set of values of *feature variables* as accurately as possible. Typical application scenarios in medicine include knowledge discovery and decision support.

Our tool implements the common *supervised learning* approach, where a model is created from a *training set*. It focusses on *classification* tasks where target variables are categorical and values of the target variable are called *classes* [20]. To create privacy-preserving prediction models, our tool implements supervised learning from anonymized data. To maximize the performance of the resulting models it utilizes the optimization procedures provided by ARX to produce anonymized output data that is suited for this purpose.

**Fig. 1:**
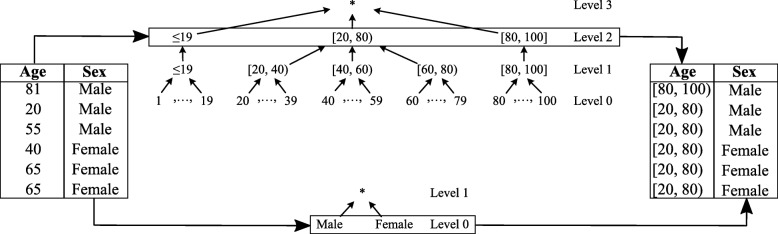
Example of attribute transformation based on generalization hierarchies. Values of the attributes “age” and “sex” are transformed using level 2 and level 0, respectively, of their associated hierarchies

At its core, ARX utilizes user-defined generalization *hierarchies* to transform data. A simple example is shown in Fig. 1. As can be seen, generalization hierarchies store the original attributes’ values in the leaf nodes while inner nodes contain generalized representations of the values from the leaf nodes of the according subtree. When a hierarchy is used to transform the values of an attribute, all values are replaced by the corresponding inner nodes on a given *level* of the hierarchy. In the example, values of the attribute “age” are transformed into age groups by replacing them with the corresponding generalized values on level 2 of the hierarchy, while values of the attribute “sex” are left as-is (which corresponds to “transforming” them to level 0 of the hierarchy). In an abstract sense, the anonymization process implemented by ARX basically produces all possible output datasets by applying all possible combinations of generalizations to the input dataset. For each possible output, two parameters are measured: (1) privacy protection, and (2) data utility. After this process, ARX returns the transformed dataset that satisfies pre-defined privacy protection levels and which is most useful. In practice, ARX implements a wide range of pruning strategies and optimization techniques to avoid needing to analyze all possible output datasets (see, e.g. [19, 21]). Moreover, ARX supports further transformation techniques which are implemented by extending the basic anonymization process outlined in this paragraph. Furthermore, privacy protection as well as data utility can be measured using different models. We will briefly introduce the most important methods used in this article in the remainder of this section.

#### Privacy models

In ARX, privacy models are used to specify and quantify levels of protection. The methods for creating privacy-preserving prediction models presented in this article are compatible with all privacy models currently implemented by ARX (an overview is provided on the project website [22]). In this paper, we will use the following models to showcase our solution: (1) *k-anonymity*, which protects records from re-identification by requiring that each transformed record is indistinguishable from at least *k*−1 other records regarding attributes that could be used in linkage attacks [15], (2) *differential privacy* which guarantees that the output of the anonymization procedure is basically independent of the contribution of individual records to the dataset, which protects output data from a wide range of risks [23, 24], and (3) a *game-theoretic model* which employs an economic perspective on data re-identification attacks and assumes that adversaries will only attempt re-identification in case there is a tangible economic benefit [25, 26].

#### Utility models

ARX supports a wide range of models for quantifying (and hence optimizing) the utility of output data. To optimize output towards suitability as a training set for prediction models, we have implemented the method by Iyengar [27]. The basic idea is to distinguish between the removal of *structure* and the removal of *noise* by measuring the heterogeneity of values of class attributes in groups of records that are indistinguishable regarding the specified feature variables. For instance, if the age of individuals and the occurrence of a certain disease exhibits a strong correlation, the relationship between these two attributes is most likely best captured by adequate age groups instead of more granular data. In prior work, we have already described a basic implementation of the approach [18]. However, the implementation had several important limitations, which resulted from the compressed internal data representation used by ARX [19]: (1) it only supported one class variable, (2) it required that class variables were addressed by a privacy model, and (3) it required that no transformations were applied to target variables. To overcome these limitations we had to rewrite major parts of the internals of the software and the resulting utility model is now the most complex model supported. Finally, we also had to develop and implement a specialized *score function* with proven mathematical properties to support differential privacy [24].

#### Transformation models

**Fig. 2:**
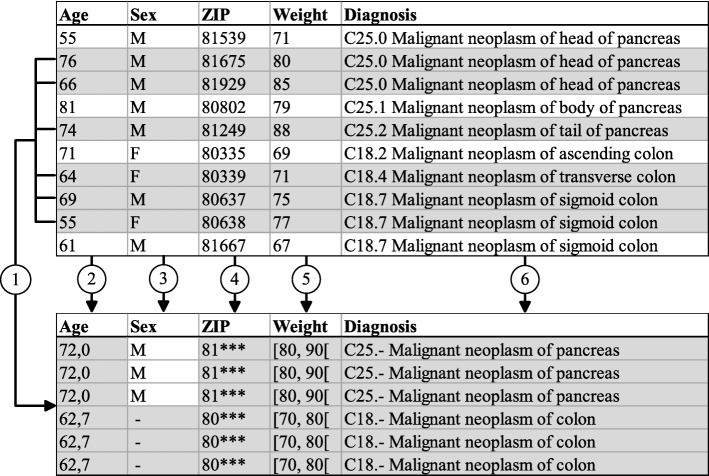
Example of different transformation schemes used in data anonymization. 1: Sampling, 2: Aggregation, 3: Suppression, 4: Masking, 5: Categorization, 6: Generalization

Based on the generic mechanism described above, ARX provides support for a wide range of transformation techniques. Different methods for transforming data can also be used in combination. Typically, this is done to preserve as much output data utility as possible and to preserve important schematic properties of data, such as the data types of variables. Figure 2 shows an example of the different methods supported: (1) *Random sampling* is a common method to reduce the certainty of attackers about the correctness of re-identifications. It is also a major building block of differential privacy in ARX [24]. (2) *Aggregation* is a method where sets of numeric attribute values are transformed into a common aggregated value. (3) *Suppression* means that values are simply removed from a dataset, which may be applied on the cell-, record- or attribute-level. (4) *Masking* is a method where individual characters are removed. (5) *Categorization* means that continuous variables are mapped to categories. (6) *Generalization* is a method where attribute values are replaced by less specific values based on user-defined generalization hierarchies or classifications, such as the International Classification of Diseases [28].

In the output dataset shown in Fig. 2, the risk of a record being re-identified correctly is not higher than 33.3% (3-anonymity). In addition, the anonymization procedure fulfills (*ε*,*δ*)-differential privacy with *ε*≈0.92 and *δ*≈0.22, under the assumption that all changes other than sampling have been implemented using a data-independent transformation method [24]. While support for the transformations utilized in the example is provided out-of-the-box by ARX, implementing evaluation methods for prediction models trained on this data needs careful attention, as we will describe in the next section.

#### Classification models

**Fig. 3:**
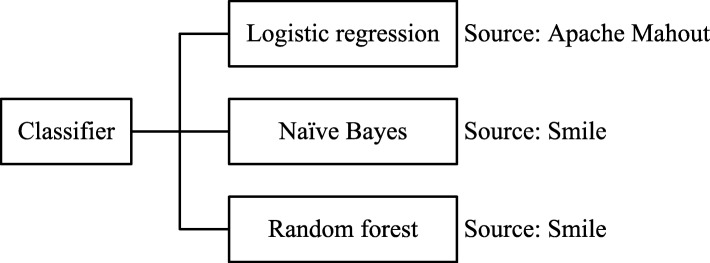
Classification models supported by the current implementation. A generic wrapper has been developed to encapsulate the implementation specifics of different machine learning libraries

To enable users to assess the performance of different types of prediction techniques, we implemented a generic interface to prediction models and integrated three methods as is shown in Fig. 3: (1) *Logistic regression*, where the relationship between the feature variables and the target variable is expressed as a linear model which is transformed using a logarithmic function [20]. Since support for this model was already established in previous work, we only had to make minor adjustments to integrate it with the new interface. (2) *Naïve Bayes* [29], which makes strong (hence naïve) assumptions about the independence of the distributions of the feature variables based on Bayes’ theorem. The only dependency is assumed to exist between the target variable and each of the feature variables. Predictions are made by simply calculating the posterior probabilities of each of the classes using the prior probability of the feature vector. (3) *Random forest* [30], which belongs to the class of *ensemble learning methods*. This means that the predictions of multiple models are combined into a single prediction. The individual models are decision trees generated from independently sampled training data by selecting a random subset of the features at each split in the learning process.

We tested a wide range of implementations that are compatible with ARX’s license and decided that we need to rely on different frameworks to integrate scalable implementations of different techniques. For this reason, we had to create a common interface already mentioned above to abstract away the details of specific implementations. We integrated logistic regression from Apache Mahout [31] and both naïve Bayes and random forest from Smile [32].

### Assessing prediction performance

#### Preprocessing training data

The creation of prediction models typically involves the process of reviewing models and iteratively refining parameters to achieve optimal performance. This requires metrics for performance assessment. A commonly used method is to calculate performance measures using *k-fold* cross-validation [33]. In this process, the records of a dataset are first divided randomly into *k* partitions of equal size, which are then iteratively analyzed by using each of the *k* partitions as evaluation and all other partitions as training data. This process yields *k* results which are combined to derive an overall estimate of the model’s performance.

**Fig. 4:**
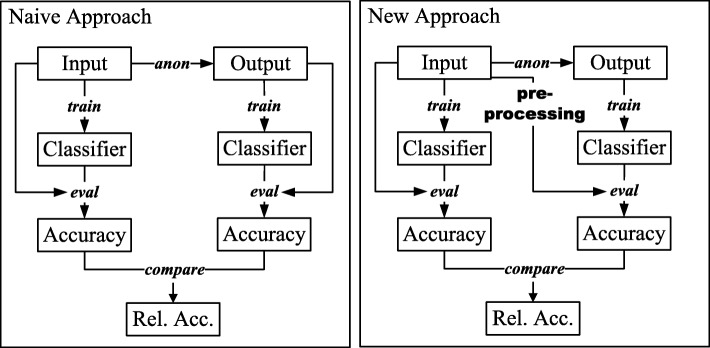
Different approaches for measuring the relative performance of a privacy-preserving classifier. Our tool implements a method that makes sure that the performance of prediction models can be expressed relative to the performance of models trained on unmodified data

When classification models are built from anonymized data, it needs to be evaluated how anonymization has affected their performance. This cannot be implemented “naively” by comparing the results of performing *k-fold* cross-validation on the anonymized data and of performing *k-fold* cross-validation on input data. Instead, a classifier must be built from transformed output data in such a way that the model is able to make predictions based on features which have not been transformed. As a result, the model can be evaluated using unmodified input data to obtain relative performance estimates [34]. This can be achieved by implementing a preprocessing step which transforms a given set of previously unknown features in the same manner in which the anonymized training data has been transformed before passing it to the classifier to make predictions [35]. Figure 4 visually contrasts both approaches. It can be seen that in the naive approach two classifiers are built from two different datasets (input and output), evaluated against these datasets and then their accuracy is compared to derive a relative performance. In our tool, the second classifier is built from output data but evaluated on (preprocessed) input data to obtain comparable results for both models.

Our tool creates privacy-preserving models by training them on anonymized data. This results in the challenge that the prediction models created can only be applied to data that has been transformed in the same way as the anonymized training dataset. Thus, we had to ensure that the resulting prediction models are able to interpret features from output data as well as input data correctly. This is challenging when the domain of attribute values is not preserved during anonymization, as in these cases, the input contains values which are not present in the output and thus the classifier would have to be evaluated with values which it has not seen during training. As a solution, we implemented a preprocessing step that accounts for the different types of transformations supported (see beginning of this section).

Whether the preprocessing step needs to be applied to a specific variable depends on the type of the variable and the transformation method utilized. Table 1 shows an overview. “N/A” indicates that the transformation method cannot be used for variables of the according type. For instance, aggregation is typically only applied to numeric attributes. It can be seen that for all types of suppression (cell, attribute, record), random sampling as well as aggregation, evaluation data does not have to be preprocessed. The reason is that the domain is being preserved during transformation. With all remaining transformation schemes, data needs to be preprocessed before handing it to the classifier for evaluation. As can be seen, preprocessing only needs to be performed for attribute values that have been generalized or categorized. In both cases, this can be implemented by applying the same generalization hierarchies or categorization functions to input data that have also been used to anonymize the training dataset. During the evaluation process this is performed automatically as all relevant information on how input data has been transformed is known to the software. For the purpose of utilizing the output data generated by ARX to build a privacy-preserving prediction model outside of the software, according export functionalities (e.g. for hierarchies) are provided.

**Table 1:** Overview of transformation schemes and their preprocessing requirements

Transformation scheme	Preprocessing required	
	Numeric attributes	Categorical attributes
Cell suppression	No	No
Attribute suppression	No	No
Record suppression	No	No
Generalization	Yes	Yes
Categorization	Yes	N/A
Aggregation	No	N/A
Random sampling	No	No

#### Performance assessment

All implemented classification models are able to handle multinomial classification tasks, where the target variables need not be dichotomous. The main reason behind this design decision is that we wanted our methods to integrate seamlessly with the remaining functionalities of ARX, without imposing any major restrictions. However, assessing the performance of multinomial classifiers is non-trivial and subject of ongoing research [20]. Our previous implementation therefore only supported very rudimentary performance measurements [18]. One method to overcome this limitation is the *one-vs-all* approach, in which the performance of a *n-nomial* classifier is assessed by interpreting it as a collection of *n* binomial classifiers, each of which is able to distinguish one selected class from all others.

We decided to implement this method as it is simple and enables utilizing typical parameters for prediction performance. Our implementation currently supports the following measures: (1) *sensitivity*, also called *recall* or *true positive rate*. (2) *Specificity*, also called *true negative rate*. (3) The *Receiver Operating Characteristic (ROC)* curve, which plots the true positive rate (i.e. the sensitivity) for a single class against the false positive rate (1-specificity) [36]. The ROC curve shows the trade-off between sensitivity and specificity for every possible cut-off for a prediction, i.e. any increase in sensitivity will be accompanied by a decrease in specificity. (4) The *Area Under the ROC Curve* (ROC AUC), which summarizes the ROC performance of a classifier and which is equivalent to the probability that the classifier will assign a higher score to a randomly chosen positive event than to a randomly chosen negative event [36]. (5) The *Brier score*, which measures the mean squared distance between predicted and actual outcomes [37].

In addition to the models described previously, we always evaluate the performance of the *Zero Rule (0-R) algorithm*, which ignores the feature variables and simply always returns the most frequent class value. The performance of this simplistic “prediction model” is frequently used as a realistic baseline for assessing the performance of more sophisticated machine learning algorithms. In our tool, the performance of privacy-preserving models is reported in absolute terms as well as relative to baseline (0-R) and the selected classifier, both trained on unmodified input data.

As an additional measure specific to our application scenario, we implemented the *skill score*, which quantifies the relative accuracy of a classification model over some reference accuracy [38]. In our case, the relative accuracy is the accuracy of the classification model built from anonymized data over the accuracy of the model built from original data. Typically, the accuracy is represented by a metric such as the Brier score, leading to the following definition:

$$Brier~skill~score = 1- \frac{Brier_{anonymized}}{Brier_{original}}  $$

A skill score of zero means that the Brier scores for models built on output and input data are equal. If the score is in the range ]0,1] then the model built on output data performed better and if it is in the range [−*∞*,0[, the model trained on the original data performed better.

## Results

### Interfaces for end users and applications

ARX’s views and interfaces for data anonymization and privacy risk analysis have been described in previous publications [19, 39] and are also explained in depth on the project website [22]. Here, we will focus on the views and interfaces provided for analyzing the performance of prediction models. All methods described in the previous sections have been implemented into the Graphical User Interface (GUI) and they are also available via the software’s comprehensive Application Programming Interface (API).

**Fig. 5:**
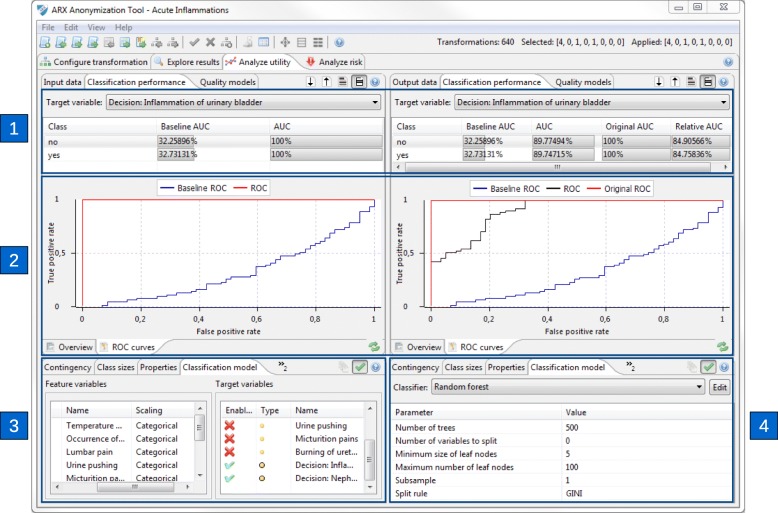
Screenshot of the view implemented for assessing the performance of privacy-preserving prediction models. Area 1: Comparison of basic performance parameters, Area 2: ROC curves for models built with original and anonymized data, Area 3: Selection of feature and class variables, Area 4: Selection and configuration of model parameters

Figure 5 shows a screenshot of the graphical interface in which methods for configuring prediction models as well as for assessing their performance have been implemented. Areas 1 and 2 can be used to graphically assess the performance of privacy-preserving models. Both views are available side-by-side for input data and output data to allow for visual comparisons. They show basic performance parameters and ROC curves for models built with original and anonymized data, respectively. Areas 3 and 4 can be used to select target variables as well as feature variables and to configure model types and their parameters.

### Case studies

In this section, we will present three case studies to illustrate our solution and to show its practical applicability. For this purpose, we have selected three datasets to build different types of models for different biomedical prediction tasks. We have deliberately selected datasets that are challenging to anonymize as they contain a small number of records (between 120 and 1473). We will use the visualizations provided by ARX to discuss the utility and privacy protection provided by the resulting models. In all cases, we measured execution times for data anonymization as well as model building and evaluation of not more than a few seconds on commodity hardware.

#### Case study 1: acute inflammation of the urinary system

In the first case study, we used a dataset containing 120 records that were originally collected for testing expert systems. The task is to diagnose two diseases of the urinary system: acute inflammation of the bladder and acute nephritises. The dataset contained nine numeric and binary attributes, two of which represented the target classes. More details can be found in the original publication [40] and the publicly available version of the dataset [41]. As a privacy model we used *k*-anonymity, which protects the records in the training set from re-identification. We used common parameterizations of 5≤*k*≤25 and random forests as prediction models. Data was transformed using aggregation, generalization and record suppression.

**Fig. 6:**
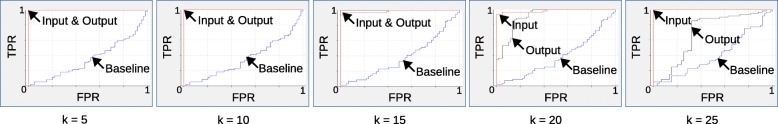
ROC performance in the case study using k-anonymous data for training random forests on the acute inflammation dataset. The False Positive Rates (FPR) and True Positive Rates (TPR) are plotted against the x-axes and y-axes, respectively. It can be seen that data anonymization had a negative impact on the performance of the resulting prediction models only for *k*≥15

Figure 6 shows the results obtained for one of the two target variables (inflammation of urinary bladder). For comparison, the blue line shows the performance achieved when always returning the most frequent class attribute (0-R). In the first two plots, the ROC of models trained on unmodified training data and anonymized data is identifical. We measured a relative ROC AUC (relative to the trivial classifier and to the performance of models trained on input data) of 100% for *k*=5 and *k*=10 and *k*=15. For higher values of *k*, performance dropped to 87.72% for *k*=20, 48.37% for *k*=25. The Brier skill scores changed from 0 to 0.08, −0.78,−1.25 and −4.05. For *k*≤20, which offers a very high degree of protection [42], the resulting privacy-preserving models exhibited high prediction power.

**Fig. 7:**
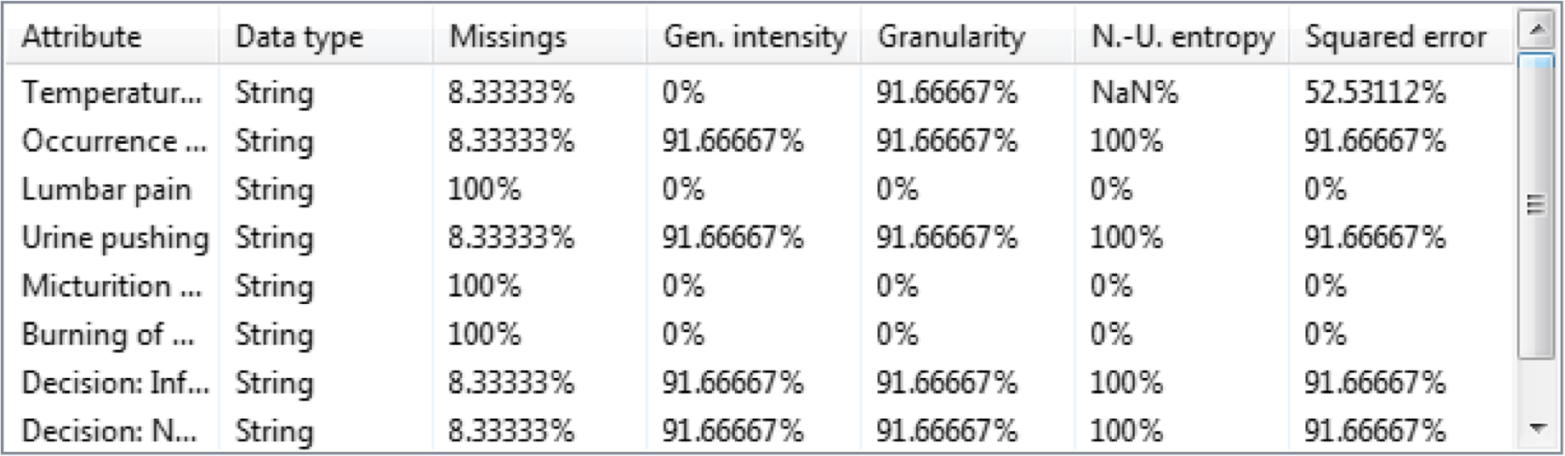
Automated dimensionality reduction performed by ARX starting from *k*=15 when anonymizing the acute inflammation dataset. For larger values of *k*, ARX performs automated dimensionality reduction during data anonymization. By comparing the results with the ROC curves in Fig. 6 it can be seen that the removal of three out of six feature variables had only a minor impact on prediction performance

When anonymizing data, ARX may determine that an optimal balance between privacy protection and output data utility is achieved by completely generalizing (and thereby actually removing) one or multiple attributes. This can be interpreted as automated dimensionality reduction or feature selection. Figure 7 shows that for *k*=15 three out of six feature variables were removed (Missings = 100%). From the results presented in the previous paragraph we can see that this had only a minor impact on prediction performance, which implies that the variables that have been removed are not predictive for the target variable. If the target variable needs to be protected from inference attacks, this information can be used as an indicator that the variables that have been removed may not needed to be transformed at all.

**Fig. 8:**
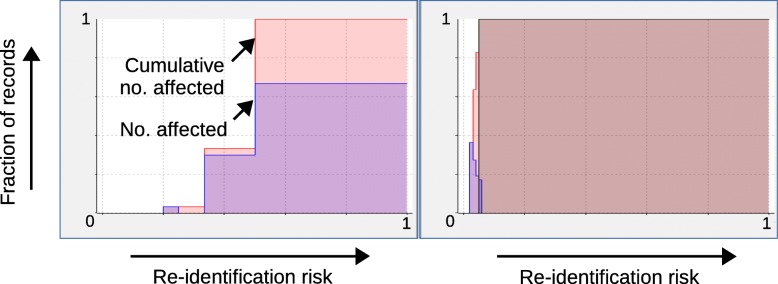
Impact of data anonymization on re-identification risk profiles for the acute inflammation dataset. As can be seen, *k*-anonymity with *k*=15 significantly reduced the risk of re-identification for all records in the dataset

Finally, Fig. 8 shows re-identification risk profiles provided by ARX (cf. [39]). A risk profile summarizes the risks of all records in a dataset, by associating each possible risk level with the relative number of records which are affected. It can be seen that *k*-anonymity with *k*=15 significantly reduced the risk of re-identification for all records in the dataset, highlighting the high degree of privacy protection that can be achieved with negligible effects on prediction performance.

#### Case study 2: breast cancer cytopathology

In the second case study, we utilized a dataset which contained 699 records collected by the University of Wisconsin Hospitals to study methods for predicting the malignancy of breast tissue from cytopathology reports. It contained 10 numeric and binary attributes, one of which represented the target class (malignant or benign tissue). The dataset and further details are available online [41].

**Fig. 9:**
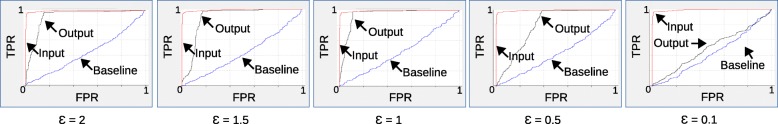
ROC performance in the case study using differential privacy for training logistic regression models to predict the malignancy of breast tissue. The False Positive Rates (FPR) and True Positive Rates (TPR) are plotted against the x-axes and y-axes, respectively. It can be seen that data anonymization had a significant impact on prediction performance, but acceptable accuracy could still be observed for *ε*≥1

For privacy protection, we utilized (*ε*,*δ*)-differential privacy with *ε*∈{2,1.5,1.0,0.5,0.1} and *δ*=10^−3^. We used logistic regression as modeling technique. Implementing differential privacy requires randomization and we therefore report on the best model obtained from five anonymization processes performed for each parameterization. Data was transformed using random sampling, categorization, generalization and record suppression. The results are shown in Fig. 9.

As can be seen in the figure, prediction performance decreased with decreasing values of epsilon, which was to be expected as the degree of privacy protection increases when epsilon decreases. Moreover, the results confirm prior findings which indicated that a value of about *ε*=1 is an optimal parameterization for the differentially private anonymization algorithm implemented by ARX [24]. Furthermore, we studied the effect of randomization on the stability of the performance of the models created. The prediction model trained on unmodified input data achieved a ROC AUC of about 99.2%. For the five models created with *ε*=1 we measured a ROC AUC of between 85.8% and 92.27% (88.28% on average) which equals a relative ROC AUC of between 61.63% and 83.96% (74.80% on average) compared to baseline performance and the model trained on unmodified data. The Brier skill score varied between -1.38 and -3.45 (-2.66 on average), which is quite good considering the high degree of privacy protection provided.

**Fig. 10:**
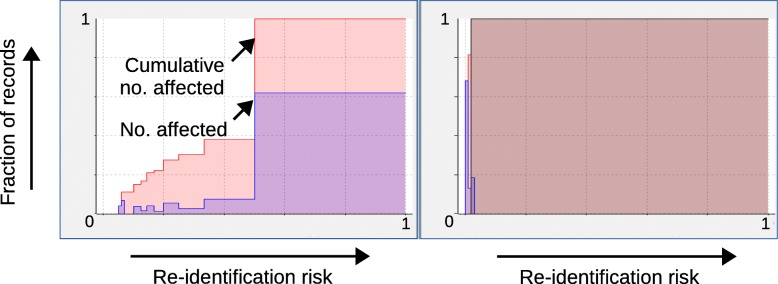
Impact of data anonymization on re-identification risk profiles for the breast cancer dataset. As can be seen, the differential privacy model with *ε*=1 resulted in the strongest reductions to re-identification risks of all models used in the case studies

Finally, Fig. 10 shows the risk profiles provided by ARX for the best model obtained using *ε*=1. As can be seen, re-identification risks were reduced to an extent even larger than in the previous case study. Moreover, we also found that ARX performed significant dimensionality reduction and that malignancy was basically predicted from a single attribute (bland chromatin).

#### Case study 3: use of contraceptive methods

In the third case study, we utilized a dataset consisting of 1473 records from the 1987 National Indonesia Contraceptive Prevalence Survey to predict the contraceptive method used of women based on their demographic and socio-economic characteristics. The dataset contained 10 numeric, categorical and binary attributes, one of which represented the target class (type of contraceptive method used). More details can be found in the original publication [43] and the dataset is available online [41].

For privacy protection, we employed an innovative game-theoretic method that works on the assumption that adversaries will only attack a dataset (or prediction model) if there is a tangible economic benefit. For parameterizing the method, we followed the proposal by Wan et al. [25]: the cost for the adversary of trying to re-identify an individual was set to $4 (a number that has been derived from the costs of obtaining detailed personal information online) and the monetary benefit of including a record into the training set was assumed to be $1200 (this number was derived from an analysis of grant funding received and data shared by the Electronic Medical Records and Genomics (eMERGE) Network [44], which is funded by the National Institute of Health (NIH)).

We considered a single free parameter *G*, which specified the monetary gain of the adversary in case of successful re-identification and, at the same time, the monetary loss for the data controller for each successfully re-identified record. By varying this single parameter we were able to investigate a wide variety of scenarios, in which either the data controller or the adversary was at an advantage. For prediction, we used Naïve Bayes classifiers. Data was transformed using categorization, generalization as well as cell and record suppression.

**Fig. 11:**
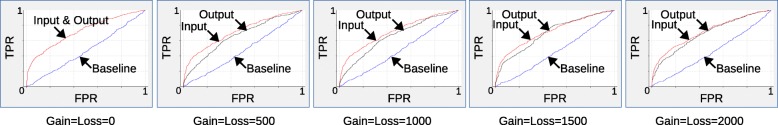
Impact of data anonymization on prediction performance in the contraceptive methods case study. The False Positive Rates (FPR) and True Positive Rates (TPR) are plotted against the x-axes and y-axes, respectively. As can be seen, data anonymization using the game-theoretic model had only a very minor impact on prediction accuracy

Overall, as can be seen in Fig. 11, we found that anonymizing the dataset with *G*=0,500,1000,1500 and 2000 had only a very limited impact on the performance of the resulting privacy-preserving prediction models. Models trained on unmodified input data achieved a ROC AUC of 71.82%. We were not able to observe a relationship between privacy parameters and the prediction performance of the privacy-preserving models. The reason is that the game-theoretic model contains an implicit data quality model that does not directly reflect the suitability of data for training prediction models. We measured a relative ROC AUC between 77.33% and 100% (90.35% on average) and Brier skill scores between -0.04 and 0 (-0.02 on average). Analogously to the other studies, we observed a significant reduction of re-identification risks.

## Discussion

### Comparison with prior work

Early work has suggested that anonymization destroys the utility of data for machine learning tasks [45]. Many methods for optimizing anonymized data as a training set for prediction models have since been developed. They show that this is not actually true. Initially, these methods focused on simple anonymization techniques, such as *k-anonymity*, and simple prediction models, such as decision trees and on applications in distributed settings [35, 46]. As a result of these developments, evaluating (novel) anonymization methods by measuring the usefulness of output data for predictive modeling tasks has become a standard practice in academia [47, 48]. More recently, a broader spectrum of prediction and privacy models has been investigated. Some authors proposed general-purpose anonymization algorithms to optimize prediction performance. While most of these algorithms have been designed in such a way that the resulting anonymized data is guaranteed to provide a degree of protection based on specific privacy models only [49, 50], they allow for any type of prediction model to be used. In contrast, in other works, privacy-preserving algorithms for optimizing the performance of specific prediction models were developed [51, 52]. Many recent studies focused on sophisticated models, such as support vector machines [51, 53, 54] and (deep) neural networks [55–57]. More complex and comprehensive privacy models have also received significant attention. In particular, the differential privacy model was investigated extensively [53, 55, 56, 58–62]. It is notable, that among these more modern approaches, a variety has focused on biomedical data [56, 57, 60]. We note, however, that these developments originate from the computer science research community and if the developed algorithms are published, then typically only in the form of research prototypes.

In parallel, several practical tools have been developed that make methods of data anonymization available to end-users by providing easy-to-use graphical interfaces. Most notably, *μ*−*ARGUS* [63] and *sdcMicro* [64] are tools developed in the context of official statistics, while ARX has specifically been designed for applications to biomedical data [19]. *μ*-ARGUS and sdcMicro focus on the concept of *a posteriori disclosure risk control* which is prevalent in the statistics community. In this process, data is mainly transformed manually in iterative steps, while data utility, usefulness and risks are monitored continuously by performing statistical analyses and tests. ARX implements a mixture of this approach and the *a priori disclosure risk control* methodology. This means that data is anonymized semi-automatically. In each iteration, the data is sanitized in such a way that predefined thresholds on privacy risks are met while the impact on data utility is minimized. A balancing is performed by repeating this process with different settings, thereby iteratively refining output data. This approach has been recommended for anonymizing health data (see, e.g. [7, 12] and [13]) and it enables ARX to support an unprecedentedly broad spectrum of techniques for transforming data and measuring risks. All three tools provide users with methods for assessing and optimizing the usefulness of anonymized data for a wide variety of applications. ARX is, however, the only tool providing support for privacy-preserving machine learning.

### Limitations and future work

Currently, our tool only supports three different types of prediction models, i.e. logistic regression, naïve Bayes and random forest, for which we could find scalable implementations that are compatible to ARX in terms of their technical basis and licensing model. However, further approaches, e.g. C4.5 decision trees and support vector machines, have also received significant attention in the literature (see e.g. [49–51, 53, 54, 58, 60, 62]). In future work, we plan to extend our implementation accordingly. Moreover, choosing the right type of prediction model for a specific dataset and task is challenging, as there are no general recommendations [20]. Therefore, benchmark studies are often performed, in which the results of different models are experimentally compared for a specific dataset using a complex process involving the separation of data into training sets, evaluation sets and validation sets [65]. In future work, we plan to extend our implementation to support such benchmark studies for privacy-preserving models as well.

In this article we have focused on transformation techniques supported by ARX for which a preprocessing step can be implemented by applying a known transformation function to features (see “Preprocessing training data” section). The software, however, also supports transformation approaches where it is not clear how a given feature must be transformed to match the representation used for training purposes. Local generalization is an important example. In this case, the same attribute value can be transformed to different generalized representations in different records of the training set. When providing features to the model to make predictions, it is therefore unclear how the values of such attributes must be generalized. One approach to overcome this challenge is to apply all possible transformations and to then analyze which transformation results in the prediction with the highest confidence. However, this involves a high degree of complexity and we therefore plan to develop more scalable approaches in the future.

Finally, our current implementation focuses on classification tasks. In future work, we plan to provide support for further learning and prediction tasks that are of specific importance to medical research. Important examples include regression and time-to-event analysis [20].

## Conclusions

In this paper, we have presented a comprehensive tool for building and evaluating privacy-preserving prediction models. Our implementation is available as open source software. We have further presented three case studies which show that, in many cases, a high degree of privacy protection can be achieved with very little impact on prediction performance. Our tool supports a wide range of transformation techniques, methods for privacy protection and prediction models. The methods supported are particularly well suited for applications to biomedical data. Notably, the truthful transformation methods implemented prevent implausible data from being created (e.g. combinations or dosages of drugs which are harmful for a patient) [66]. Moreover, methods of privacy preservation have been implemented in a way that is relatively easy to explain to ethics committees and policy makers, as they basically rely on the intuitive idea of hiding in a crowd [24]. To our knowledge, ARX is the only publicly available anonymization tool supporting a comprehensive set of methods for privacy-preserving machine learning in an integrated manner.

## Availability and requirements

- **Project name**: ARX Data Anonymization Tool
- **Project home page**: https://arx.deidentifier.org/
- **Operating system(s)**: Platform independent
- **Programming language**: Java
- **Other requirements**: Java 1.8 or higher
- **License**: Apache License, Version 2.0
- **Any restrictions to use by non-academics**: No

## Data Availability

The datasets used during the current study are available from the corresponding author on reasonable request

## Abbreviations

0-R: Zero rule
API: Application programming interface
AUC: Area under the curve
eMERGE: Electronic medical records and genomics
GUI: Graphical user interface
HIPAA: U.S. health insurance portability and accountability act
NIH: National institute of health
ROC: Receiver operating characteristic
